# *Enterococcus burkinafasonensis* sp. nov*.* isolated from human gut microbiota

**DOI:** 10.1016/j.nmni.2020.100702

**Published:** 2020-06-01

**Authors:** N. Gouba, E.K. Yimagou, Y. Hassani, M. Drancourt, M. Fellag, M.D. Mbogning Fonkou

**Affiliations:** 1) Research and Training Unit in Technical Sciences (UFR-ST), Nazi Boni University, Bobo-Dioulasso, Burkina Faso; 2) Aix Marseille Univ, IRD, AP-HM, MEPHI, IHU-Méditerranée Infection, Marseille, France

**Keywords:** Culturomics, *Enterococcus*, *Enterococcus burkinafasonensis*, gut, new species, taxonogenomics

## Abstract

Strain Marseille-Q0835^T^ is an aerobic, non-motile and non-spore-forming Gram-positive coccus isolated from the stools of a Burkinabe woman. In this report, we present its phenotypic description including MALDI-TOF mass spectrometry analysis and genome sequencing. Strain Marseille-Q0835^T^; 2.9768-Mb genome exhibited a 41.9 mol% G+C content and 2699 predicted genes. Considering phenotypic features and comparative genome studies, we propose the strain Marseille-Q0835^T^ as the type strain of *Enterococcus burkinafasonensis* sp. nov., a new species within the family *Enterococcaceae*.

## Introduction

Culturomics strategy is a high-throughput culturing method [1]. This strategy consists of the diversification of culture conditions and uses matrix-assisted laser desorption/ionization time-of-flight mass spectrometry (MALDI-TOF/MS) for identification, to study the human microbiota [[1], [2], [3]]. Culturomics has played a fundamental role in resolving the gaps in 16S rRNA gene-targeted metagenomics [4]. It has been reported that culturomics has contributed up to 66.2% towards updating the repertoire of isolated human bacterial and archaeal species [2]. Taxonogenomics is a concept used for the description of new species that includes phenotypic data, MALDI TOF/MS data and genome sequencing [5,6]. In this study, we report a human gut isolate representative of a novel *Enterococcus* species purposely named *Enterococcus burkinafasonensis*.

## Isolation and growth conditions

**Fig. 1:**
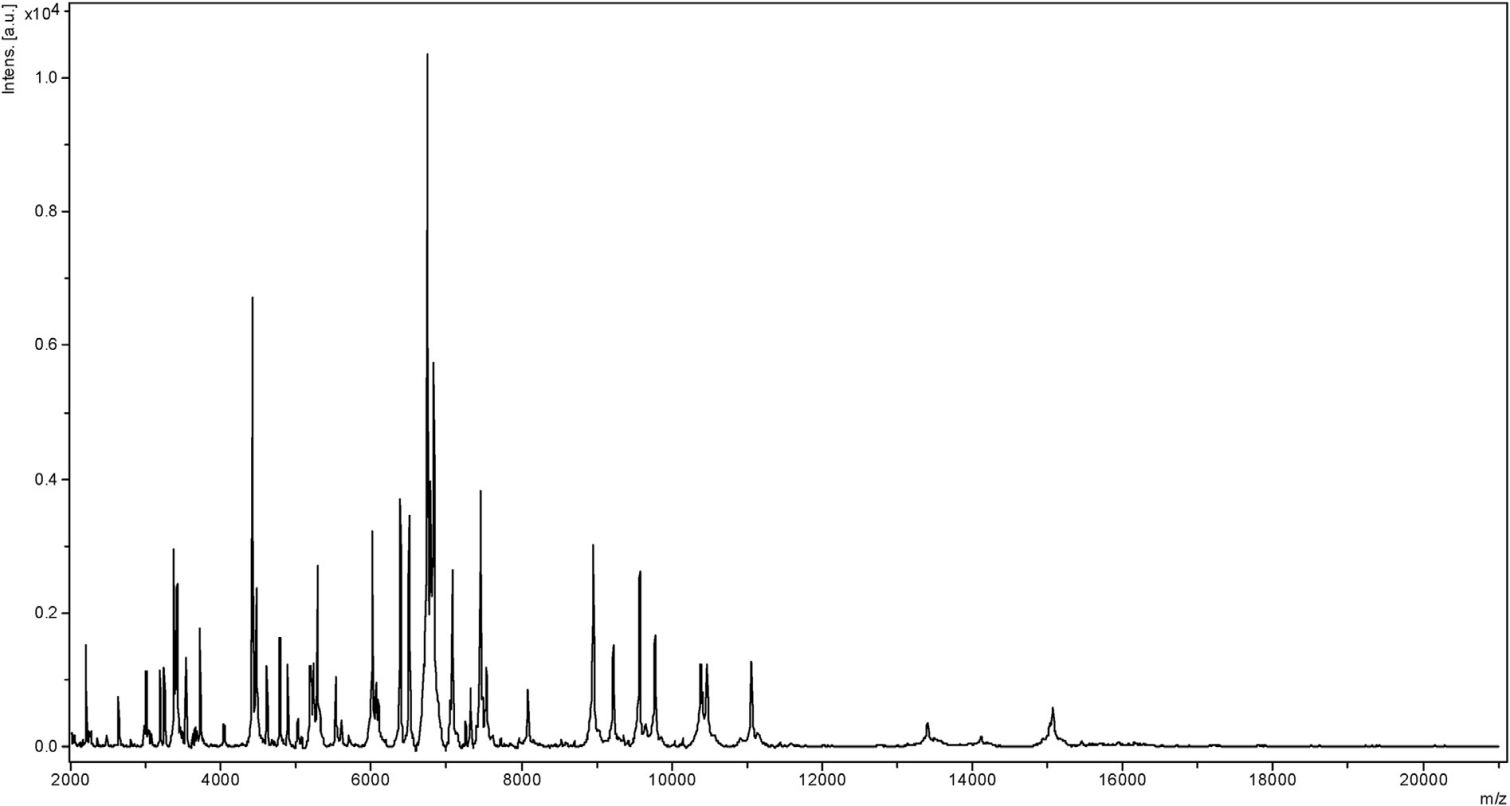
MALDI-TOF MS reference mass spectrum. Spectra from 12 individual colonies of *Enterococcus burkinafasonensis* strain Marseille-Q0835^T^ were compared and a reference spectrum was generated.

In September 2018, a fresh stool sample was collected from an apparently healthy 28-year-old Burkinabe woman who was admitted for diagnosis check-up in the Regional Tuberculosis Control Centre, Bobo-Dioulasso, Burkina Faso. A stool sample was sent to the collaborative laboratory at IHU in Marseille, France for culturomics analysis, which isolated an unidentified bacterial strain from the stool. The study was validated by the Science and Health Research Ethics Committee of Bobo-Dioulasso, under number (N/Ref.002-2018-CEIRS). The bacterium here referred to as strain Marseille-Q0835 was isolated on Columbia sheep blood agar after a 24-hour incubation under aerobic atmosphere at 37°C and pH 7.5. Purified colonies could not be identified by MALDI-TOF MS. The screening was performed on a Microflex LT spectrometer (Bruker Daltonics, Bremen, Germany), as previously described [7]. The obtained spectra (Fig. 1) were imported into MALDI Biotyper 3.0 software (Bruker Daltonics) and analysed against the main spectra of the bacteria included in the database.

## 16S rRNA gene sequencing

**Fig. 2:**
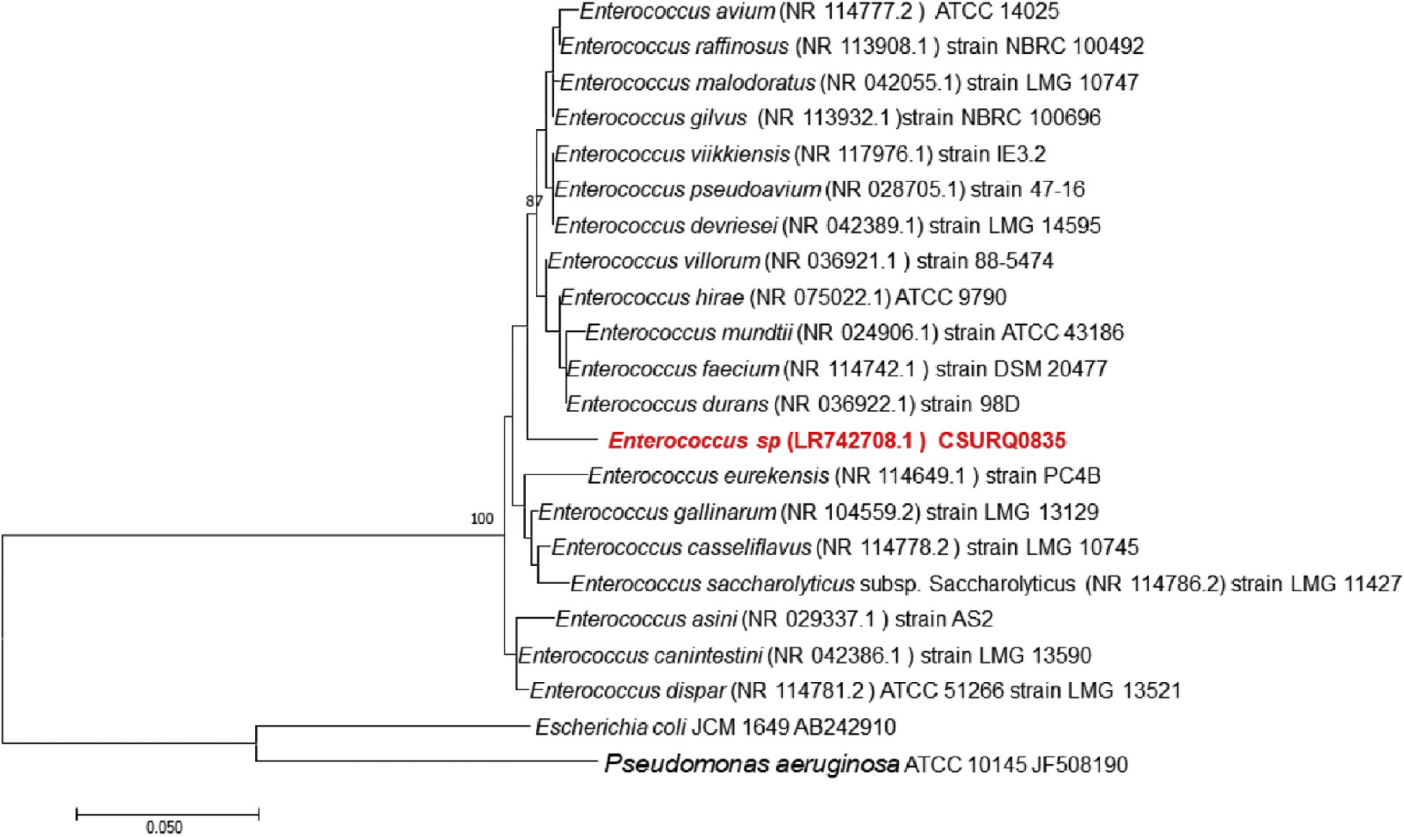
Phylogenetic tree showing the position of *Enterococcus burkinafasonensis* strain Marseille-Q0835^T^ relative to other phylogenetically close neighbours, based on the 16S rRNA gene sequences. *Pseudomonas aeruginosa* ATCC10145 and *Escherichia coli* strain JCM 1649 AB242910 are used as the outgroup. Sequences were aligned using MUSCLE, and phylogenetic inferences were obtained using maximum-likelihood method within MEGA software. Numbers at nodes are percentages of bootstrap values obtained by repeating analysis 1000 times to generate a majority consensus tree. Only bootstrap values of at least 70 were retained.

The 16S rRNA gene was sequenced in an attempt to classify this bacterium. Amplification was performed using the primer pair fD1 and rP2 (Eurogentec, Angers, France) and sequencing using the Big Dye® Terminator v1.1 Cycle Sequencing Kit and ABI Prism 3130xl Genetic Analyzer capillary sequencer (Thermofisher, Saint-Aubin, France), as previously described [8]. The 16S rRNA gene nucleotide sequences were assembled and corrected using CodonCode Aligner software (http://www.codoncode.com). BLASTn research was conducted using nucleotide databases for cross-species comparison (https://blast.ncbi.nlm.nih.gov/Blast.cgi?PROGRAM=blastn&PAGE_TYPE=BlastSearch&LINK_LOC=blasthome). The search was limited to records that include sequences from type material, and exclude uncultured/environmental sample sequences. The result showed that strain Marseille-Q0835 exhibited a 97.80% sequence identity with *Enterococcus gallinarum* strain LMG 13129 (GenBank accession number NR_104559.2), the phylogenetically closest species with standing in nomenclature (Fig. 2). We consequently classify strain Marseille-Q0835 as representative of a new species within the genus *Enterococcus*, family *Enterococcaceae*, phylum Firmicutes*.*

## Phenotypic characteristics

**Fig. 3:**
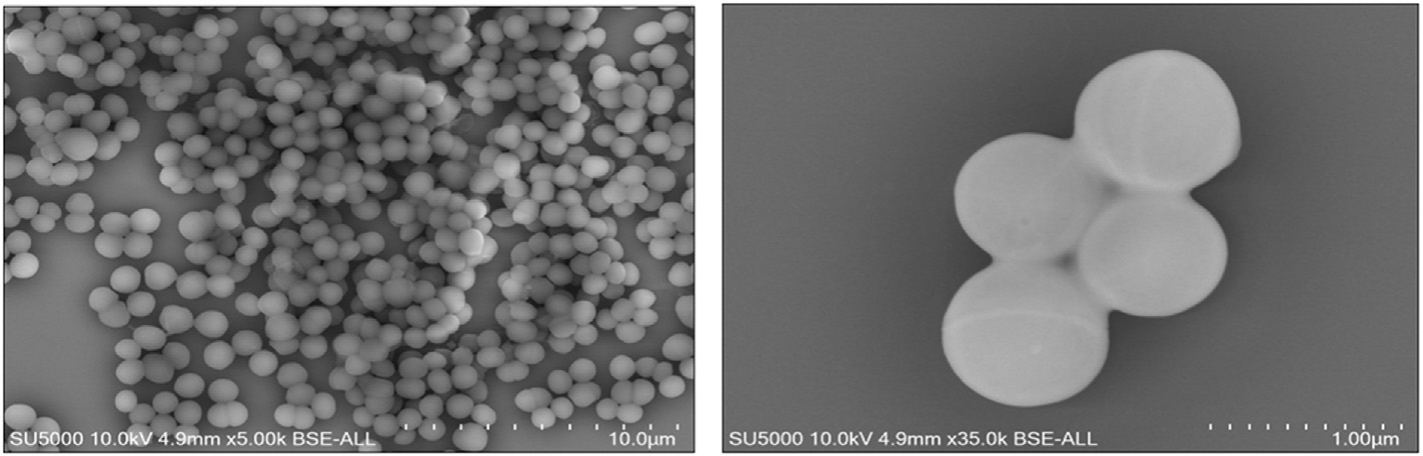
Electron micrograph of *Enterococcus burkinafasonensis* strain Marseille-Q0835^T^ was acquired with a Hitachi SU 5000 Plus tabletop scanning electron microscope.

Colonies were smooth, white with entire edges with a mean diameter of 1 mm. The bacterial cells were Gram-positive cocci, non-motile, non-spore forming with a mean diameter of 0.7 μm (Fig. 3). *Enterococcus* sp. Marseille- Q0835^T^ showed negative catalase and oxidase activities. API 50CH and API ZYM tests (bioMérieux, La Balme les Grottes, France) were performed at 37°C under aerobic conditions and the results are summarized in Table 1. Table 2 compares the characteristics of *Enterococcus* sp. nov. strain Marseille-Q0835^T^ with other bacterial species (Table 2).

**Table 1:** Phenotypic characterization of *Enterococcus burkinafasonensis* strain Marseille-Q0835^T^ based on the biochemical tests

Tests	Results
API 50 CH	
Control	
Glycerol	–
Erythol	–
d-arabinose	–
l-arabinose	+
d-ribose	+
d-xylose	–
l-xylose	–
d-adonitol	–
Methyl-βd-xylopyranoside	–
d-galactose	+
d-glucose	+
d-fructose	+
d-mannose	+
l-sorbose	–
l-rhammose	+
Dulcitom	–
Inositol	–
d-mannitol	+
d-sorbitol	–
Methyl-αd-mannopyranoside	–
Methyl-αd-glucopyranoside	–
*N*-acetylglucosamine	+
Amygdalin	–
Arbutin	+
Esculin	+
Salicin	+
d-cellobiose	+
d-maltose	+
d-lactose	–
d-melibiose	–
d-saccharose	–
d-trehalose	+
Inulin	–
d-melezitose	–
d-raffinose	–
Starch	–
Glycogen	–
Xylitol	–
Gentibiose	+
d-turanose	–
d-lyxose	–
d-tagatose	–
d-fucose	–
l-fucose	–
d-arabitol	–
l-arabitol	–
Potassium gluconate	–
Potassium 2-ketogluconate	–
Potassium5-Ketogluconate	–
API ZYM	
Alkaline phosphatase	–
Esterase (C4)	–
Esterase lipase (C8)	+
Lipase (C14)	+
Leucine arylamidase	–
Valine arylamidase	–
Cystine arylamidase	–
Trypsin	–
α-chymotrypsin	–
Acid phosphatase	–
Naphthol-AS-BI-phosphohydrolase	+
α-galactosidase	+
β-galactosidase	+
β-glucuronidase	–
α-glucosidase	–
β-glucosidase	–
*N*-acetyl-β-glucosaminidase	+
α-mannosidase	–
α-fucosidase	–

+, positive result; –, negative result.

**Table 2:** Differential characteristics of *Enterococcus burkinafasonensis* strain Marseille-Q0835, *Enterococcus timonensis* strain Marseille-P2817, *Enterococcus hirae* strain ATCC 9790, *Enterococcus gallinarum* strain NBRC 100675, *Enterococcus saccharolyticus* strain ATCC 43076, *Enterococcus casseliflavus* strain NBRC 100478 and *Enterococcus asini* strain ATCC 700915

Properties	*E. burkinafasonensis*	*E. timonensis*	*E. hirae*	*E. gallinarum*	*E. saccharolyticus*	*E. casseliflavus*	*E. asini*
	Marseille-Q0835	Marseille-P2817	ATCC 9790	NBRC 100675	ATCC 43076	NBRC 100478	ATCC 700915
Cell diameter (μm)	0.7	0.65–1.1	na	na	na	na	na
Oxygen requirement	Facultative anaerobe	Facultative anaerobe	Facultative anaerobe	Facultative anaerobe	Facultative anaerobe	Facultative anaerobe	Facultative anaerobe
Gram stain	Positive	Positive	Positive	Positive	Positive	Positive	Positive
Motility	Non-motile	Motile	Non-motile	Non-motile	na	Motile	Non-motile
Endospore formation	—	—	na	—	na	na	—
Optimum temperature for growth (°C)							
Production of:							
Alkaline phosphatase	—	+	—	na	+	na	—
Catalase	—	—	—	—	—	—	—
Oxidase	—	—	na	na	na	na	na
α-Glucosidase	—	+	na	na	—	na	na
β-Galactosidase	+	+	+	+	—	+	—
Acid from:							
N-Acetylglucosamine	+	+	+	+	+	+	+
l-Arabinose	—	—	—	+	—	+	—
d-Ribose	+	—	+	+	—	+	—
d-Mannose	+	+	+	+	na	+	—
d-Mannitol	+	+	—	+	+	+	—
d-Glucose	+	+	+	+	+	+	+
d-Fructose	+	+	+	+	+	+	+
d-Maltose	+	+	+	+	+	+	+
d-Lactose	—	+	+	+	+	+	+
G+C content (mol%)	41.9	38.46	36.9	39.80	36.90	42.40	44.70
Habitat	Human gut	Human lung	Chicken and pig intestines	Intestines of domestic fowl	Fresh broccoli	Plant material	Caecum of donkeys

## Genome sequencing

Genomic DNA was extracted using the EZ1 biorobot (Qiagen, Courtaboeuf, France) with the EZ1 DNA tissue kit and then sequenced on MiSeq technology (Illumina, San Diego, CA, USA) with the Nextera XT Paired end (Illumina), as previously described [9]. The assembly was performed with a pipeline incorporating different softwares (Velvet [10], Spades [11] and Soap Denovo [12]) on trimmed data (Trimmomatic [13]) or raw data. GapCloser was used to reduce assembly gaps. Scaffolds <800 bp and scaffolds with a depth value < 25% of the mean depth were removed [14]. The best assembly was selected by using different criteria (17 scaffolds, 19 contigs). The genome of strain Marseille-Q0835^T^ is 2.9768 Mb long with a 41.9 mol% G+C content and contains 2699 predicted genes.

**Fig. 4:**
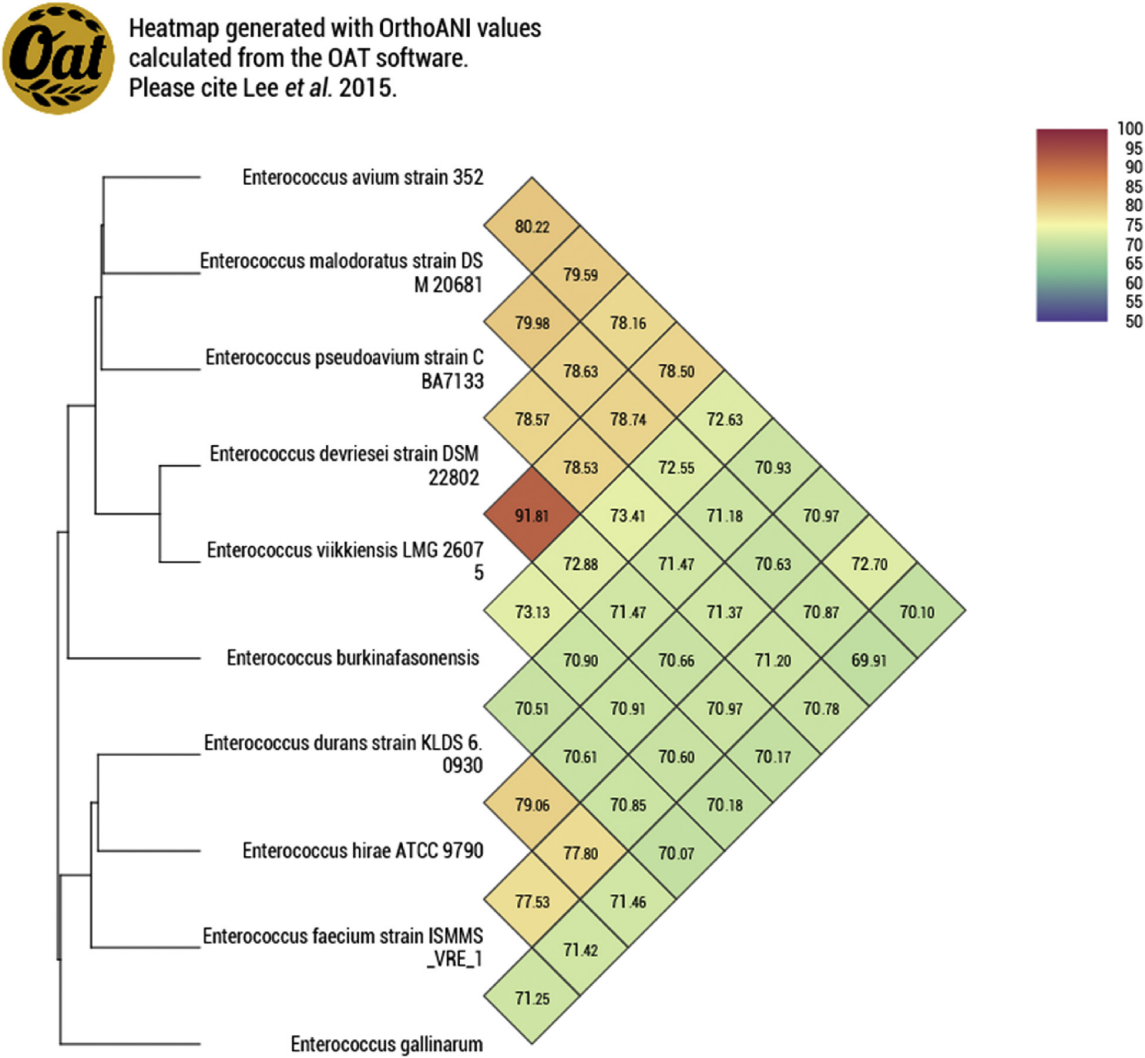
Heatmap generated with OrthoANI values calculated using the OAT software between *Enterococcus burkinafasonensis* strain Marseille-Q0835^T^ and other closely related species with standing in nomenclature.

The degree of genomic similarity of *Enterococcus* sp. Marseille-Q0835^T^ with closely related species was estimated using the OrthoANI software version 0.93.1 (https://www.ezbiocloud.net/tools/orthoani) [15]. Values among closely related species (Fig. 4) ranged from 69.91% for *Enterococcus malodoratus* strain DSM 20681 and *Enterococcus gallinarum* strain LMG 13129 to 91.81% for *Enterococcus devriesei* strain DSM 22802 and *Enterococcus viikkiensis* strain LMG 26075*.* When the isolate was compared with these closely related species, values ranged from 70.07% with *E. gallinarum* strain LMG 13129 to 73.41% with *Enterococcus pseudoavium* strain CBA7133. These values are lower than the 95% threshold used to discriminate bacterial species [15]*.*

*In silico* DNA–DNA hybridization values obtained using the GGDC version 2.0 online tool (http://ggdc.dsmz.de/ggdc.php) are reported in Table 3. For strain Marseille-Q0835^T^, these values ranged from 20% with *E. devriesei* strain DSM 22802 to 26.8% with *Enterococcus faecium* strain ISMMS VRE 1. Such values were lower than the 70% threshold recognized as delineating distinct species [16,17]*.*

**Table 3:** Digital DNA–DNA hybridization values obtained by sequence comparison of all studied genomes using GGDC, formula 2^a^

Digital DNA–DNA hybridization										
	1	2	3	4	5	6	7	8	9	10
1	100									
2	24.10% (21.8%-26.5%)	100								
3	22.40% (20.2%-24.9%)	20.00% (17.8%-22.4%)	100							
4	25.20% (22.8%-27.7%)	23.80% (21.5%-26.3%)	26.40% (24%-28.9%)	100						
5	40.60% (38.1%-43.1%)	26.80% (24.4%-29.3%)	30.00% (27.6%-32.5%)	25.60% (23.3%-28.1%)	100					
6	25.80% (23.4%-28.2%)	23.60% (21.3%-26%)	23.00% (20.7%-25.4%)	24.80% (22.5%-27.3%)	28.60% (26.2%-31.1%)	100				
7	24.40% (22.1%-26.9%)	23.00% (20.7%-25.5%)	21.30% (19%-23.7%)	23.90% (21.6%-26.4%)	23.40% (21.1%-25.8%)	25.30% (22.9%-27.7%)	100			
8	24.20% (21.9%-26.7%)	20.40% (18.2%-22.9%)	22.80% (20.5%-25.2%)	25.40% (23%-27.9%)	28.90% (26.5%-31.4%)	24.60% (22.3%-27.1%)	22.80% (20.5%-25.2%)	100		
9	23.90% (21.6%-26.4%)	22.80% (20.5%-25.3%)	22.50% (20.3%-25%)	25.10% (22.7%-27.6%)	27.80% (25.4%-30.3%)	26.40% (24%-28.9%)	27.60% (25.2%-30%)	23.80% (21.5%-26.2%)	100	
10	22.70% (20.4%-25.2%)	20.60% (18.4%-23%)	46.90% (44.3%-49.4%)	25.10% (22.8%-27.6%)	26.40% (24%-28.9%)	25.20% (22.9%-27.7%)	24.00% (21.7%-26.5%)	22.50% (20.2%-25%)	22.60% (20.3%-25.1%)	100

(1) Enterococcus avium strain 352, (2) Enterococcus burkinafasonensis strain Marseille-Q0835^T^, (3) Enterococcus devriesei strain DSM 22802, (4) Enterococcus durans strain KLDS 6.0930, (5) Enterococcus faecium strain ISMMS VRE 1, (6) Enterococcus gallinarum strain LMG 13129, (7) Enterococcus hirae strain ATCC 9790, (8) Enterococcus malodoratus strain DSM 20681, (9) Enterococcus pseudoavium strain CBA7133 and (10) Enterococcus viikkiensis strain LMG 26075.

a GGDC formula 2: (DNA–DNA hybridization estimates based on identities/high-scoring segment pair length).

**Table 4:** Description of *Enterococcus burkinafasonensis* sp. nov. strain Marseille-Q0835^T^

Type of description	New description
Species name	*Burkinafasonensis*
Genus name	*Enterococcus*
Specific epithet	*Burkinafasonensis*
Species status	sp. nov.
Species etymology	*Enterococcus burkinafasonensis* (bur.ki.na.fa.so.nen'sis, L. masc. adj. burkinafasonensis related to Burkina Faso, the name of the country where the sample was collected)
Authors	Nina GOUBA, Edmond KUETE YIMAGOU, Yasmine HASSANI, Jamal SAAD, Mustapha FELLAG, Michel DRANCOURT, Maxime Descartes MBOGNING FONKOU
Designation of the type strain	Marseille-Q0835
Strain collection number	CSURP0835
16S rRNA gene accession number	LR746132.1
Genome accession number	CADDWJ010000001.1
Genome status	Whole genome
Genome size	2.9768 Mb
GC%	41.9
Country of origin	Bobo-Dioulasso, Burkina Faso
Date of isolation	04/05/2019
Source of isolation	Human stool sample
Growth medium, incubation conditions used for standard cultivation	Growth on Columbia agar supplemented with 5% sheep's blood after 24 hours of incubation under aerobic atmosphere at 37°C and pH 7.5.
Gram stain	Positive
Cell shape	Coccus
Cell size	Mean diameter 0.7 μm
Motility	Non-motile
Sporulation	Non-sporulating
Colony morphology	smooth, white with entire edges with an average diameter of 1 mm.
Temperature range	Mesophile
Temperature optimum	37°C
Relationship to O_2_	Facultative anaerobe
O_2_ for strain testing	Anaerobiosis, microaerophilic, aerobiosis
Oxidase	Negative
Catalase	Negative

## Conclusion

Strain Marseille-Q0835^T^ exhibited a 16S rRNA gene sequence divergence <98.65%, DNA–DNA hybridization values < 70% and an OrthoANI value < 95% with its phylogenetically closest species with standing in nomenclature, together with unique phenotypic features. We formally propose strain Marseille-Q0835^T^ as the type strain of the new species named *Enterococcus burkinafasonensis*.

## Description of Enterococcus burkinafasonensis sp. nov.

Enterococcus burkinafasonensis (bur.ki.na.fa.so.nen'sis, L. masc. adj. burkinafasonensis related to Burkina Faso, the name of the country where the sample was collected). The bacterium belongs to the family *Enterococcaceae* within the phylum Firmicutes. The type strain Marseille-Q0835^T^ (CSUR P0835) was isolated after a 24-hour incubation at 37°C and pH 7.5 in an anaerobic atmosphere of a fresh stool sample collected from a 28-year-old Burkinabe woman. Colonies were smooth, white with entire edges with an average diameter of 1 mm. Bacterial cells were Gram-positive, coccus-shaped, non-motile and non-spore-forming with negative catalase and oxidase activities (see Table 4).

Using an APIZYM strip, strain Marseille-Q0835^T^ exhibits positive reaction for esterase lipase (C8), lipase (C14), naphthol-AS-BI-phosphohydrolase, α-galactosidase, β-galactosidase and *N*-acetyl-β-glucosaminidase, but negative reaction for alkaline phosphatase, esterase (C4), leucine arylamidase, valine arylamidase, cystine arylamidase, trypsin, α-chymotrypsin, acid phosphatase, β-glucuronidase, α-glucosidase, β-glucosidase, α-mannosidase and α-fucosidase. Using an API 50CH strip, positive reactions were obtained for d-galactose, d-glucose, d-fructose, l-arabinose, d-ribose, d-mannose, l-rhammose, d-mannitol, *N*-acetylglucosamine, arbutin, esculin, salicin, d-cellobiose, d-maltose, d-trehalose and gentibiose.

The strain Marseille-Q0835^T^ genome is 2.9768 Mb long, with a G-C content of 41.9%.

## Nucleotide sequence accession number

The 16S rRNA gene and genome sequences were deposited in GenBank under accession number LR742708 and NZ_CACSLH000000000, respectively.

## Deposit in culture collection

The strain Marseille-Q0835^T^ has been deposited in the French culture collection centre, Collection de Souches de l’Unité des Rickettsies (CSUR), under the number Q0835.

## Conflict of interest

None to declare.

## Funding sources

This work was funded by the 10.13039/100007356IHU Méditerranée Infection (Marseille, France) and by the French Government under the *Investissements d'avenir* (Investments for the Future) programme managed by the 10.13039/501100001665Agence Nationale de la Recherche (ANR, fr: National Agency for Research), (reference: Méditerranée Infection 10-IAHU-03).
